# Chloroplast Protein Turnover: The Influence of Extraplastidic Processes, Including Autophagy

**DOI:** 10.3390/ijms19030828

**Published:** 2018-03-12

**Authors:** Masanori Izumi, Sakuya Nakamura

**Affiliations:** 1 Frontier Research Institute for Interdisciplinary Sciences, Tohoku University, Sendai 980-8578, Japan; 2 Department of Environmental Life Sciences, Graduate School of Life Sciences, Tohoku University, Sendai 980-8577, Japan; 3 Precursory Research for Embryonic Science and Technology (PRESTO), Japan Science and Technology Agency, Kawaguchi 332-0012, Japan

**Keywords:** autophagy, chlorophagy, chloroplasts, Rubisco-containing bodies, photooxidative damage, plants, senescence, sugar starvation, ubiquitin proteasome system, vacuole

## Abstract

Most assimilated nutrients in the leaves of land plants are stored in chloroplasts as photosynthetic proteins, where they mediate CO_2_ assimilation during growth. During senescence or under suboptimal conditions, chloroplast proteins are degraded, and the amino acids released during this process are used to produce young tissues, seeds, or respiratory energy. Protein degradation machineries contribute to the quality control of chloroplasts by removing damaged proteins caused by excess energy from sunlight. Whereas previous studies revealed that chloroplasts contain several types of intraplastidic proteases that likely derived from an endosymbiosed prokaryotic ancestor of chloroplasts, recent reports have demonstrated that multiple extraplastidic pathways also contribute to chloroplast protein turnover in response to specific cues. One such pathway is autophagy, an evolutionarily conserved process that leads to the vacuolar or lysosomal degradation of cytoplasmic components in eukaryotic cells. Here, we describe and contrast the extraplastidic pathways that degrade chloroplasts. This review shows that diverse pathways participate in chloroplast turnover during sugar starvation, senescence, and oxidative stress. Elucidating the mechanisms that regulate these pathways will help decipher the relationship among the diverse pathways mediating chloroplast protein turnover.

## 1. Introduction

Chloroplasts are a type of plastid in plants and algae. In land plants, chloroplasts are present in green tissues, such as leaves, that are required for photosynthetic energy production. Within chloroplasts, thylakoid membranes contain pigments and proteins that form the light harvesting complex and electron transport chain, and the stroma contains soluble proteins that mediate the assimilation of carbon dioxide (CO_2_) via the Calvin cycle. In mature plant leaves, assimilated nutrients are largely stored in chloroplasts as photosynthetic proteins. For instance, the nitrogen in chloroplasts accounts for around 75% of the total leaf nitrogen in C3 species [1]. The CO_2_-fixing enzyme ribulose-1,5-bisphosphate carboxylase/oxygenase (Rubisco) in the stroma is especially abundant, accounting for 10–30% of the total leaf nitrogen, and constituting around half of the total soluble proteins in leaves [2,3]. Chloroplast proteins are degraded, and the amino acids and other molecules released during this process are reutilized in growth. Leaf senescence is a well-established developmental process during which chloroplast proteins are degraded en masse; the released amino acids are remobilized to generate juvenile tissues and produce seeds [4,5].

A portion of the photoassimilate is accumulated in chloroplasts as starch during the day, and is degraded at night to produce sucrose as the major source for respiratory energy production within mitochondria [6]. Since the availability of solar energy fluctuates under the ever-changing environment, plants occasionally need alternatives to sugars for producing the energy required for continuous growth. Stress conditions can also interfere with photosynthetic energy production; for instance, stomatal closure due to drought stress inhibits CO_2_ intake and thereby reduces photosynthetic activity in leaves [7,8]. Plants must metabolically produce alternative energy sources to survive under photosynthesis-limited conditions. Amino acids derived from chloroplast protein degradation via catabolic pathways can serve as alternative respiratory substrates [9].

Chloroplast protein degradation is also vital for maintaining chloroplast function, as chloroplast proteins constantly accumulate damage caused by sunlight during photosynthesis. Photoinhibition occurs when the photosynthetic apparatus is damaged by excess energy from strong visible light (with wavelengths of between 400 and 700 nm) [10,11,12,13]. Although chloroplasts cannot use ultraviolet-B (UVB; with wavelengths of between 280 and 315 nm) for photosynthesis, various macromolecules, such as proteins, lipids, and nucleotides, directly absorb UVB, which may result in cumulative damage [14]. To maintain photosynthetic activity and avoid the overproduction of reactive oxygen species (ROS) in response to sunlight irradiation, damaged components within chloroplasts must be removed.

Turnover of chloroplastic components is required for efficient nutrient recycling during plant senescence, respiratory energy production under photoassimilate-starved conditions, and quality control in individual chloroplasts under photooxidative damage. Chloroplasts contain various types of intraplastidic proteases, which are thought to have been derived from an endosymbiosed prokaryotic ancestor of chloroplasts [15,16]. Recent studies of chloroplast protein turnover have further demonstrated the contribution of extraplastidic protein degradation systems to nutrient or energy recycling and the removal of damaged proteins. In this review, we describe the extraplastidic pathways that facilitate the degradation of chloroplastic components, and compare the physiological roles of these pathways and the environmental and developmental stimuli that activate them.

## 2. Autophagic Degradation of Rubisco-Containing Bodies

Autophagy is an evolutionarily conserved process in eukaryotes whereby the cell sequesters a portion of cytoplasm, including organelles, for subsequent transport into lytic organelles [17,18,19]. During autophagy, a nascent double membrane-bound vesicle called an autophagosome encloses a portion of the cytoplasm. The outer membrane of autophagosomes then fuses with the vacuolar or lysosomal membrane to release the inner-membrane structures, referred to as autophagic bodies, into the vacuolar or lysosomal lumen for digestion. The basic mechanism of autophagosome formation was described in the budding yeast *Saccharomyces cerevisiae* through the identification of autophagy (*ATG*) genes [20].

The *ATG* genes required for the initiation or elongation of autophagosomal membranes are referred as core *ATGs* (*ATG1–10*, *12–14*, *16*, *18*), and these genes are also required for all types of autophagy [17]. Many core ATGs function in two conjugation cascades that are required for ATG8 lipidation and autophagosomal membrane elongation. ATG7 and ATG10 conjugate ATG12 to ATG5, and the resulting ATG12-ATG5 conjugate then interacts with ATG16 to form the ATG12-ATG5-ATG16 complex. ATG8 is processed by the protease ATG4. The resulting mature ATG8 is activated by ATG7, transferred to ATG3, and is eventually conjugated with phosphatidylethanolamine with the aid of the ATG12-ATG5-ATG16 complex. Orthologues of the yeast core *ATGs* are conserved in plant species [21,22,23], and studies of autophagy-deficient *atg* mutants of *Arabidopsis thaliana* show that they have similar functions [24,25,26,27,28,29,30,31,32].

**Figure 1:**
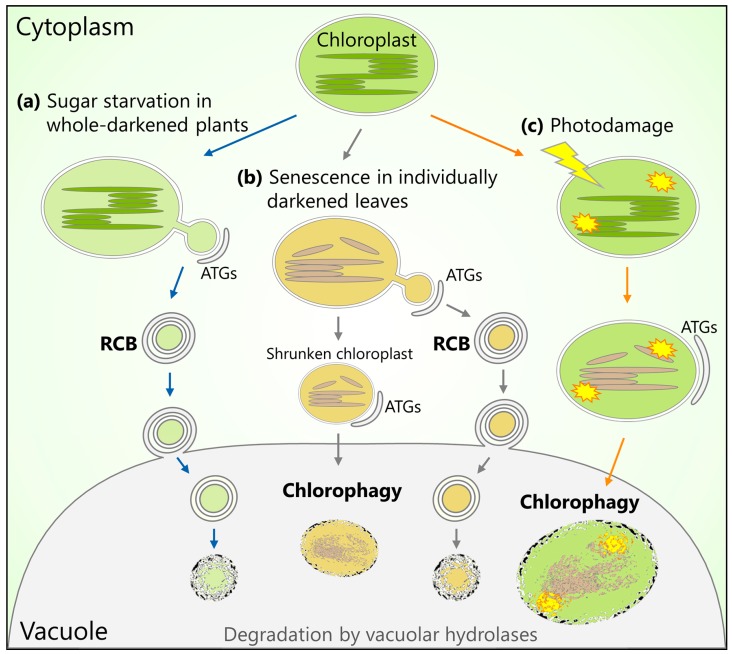
Schematic model for the Rubisco-containing body (RCB) pathway and chlorophagy forms of chloroplast-related autophagy. (**a**) When photosynthetic energy production of whole plants is impaired due to complete darkness, a portion of the chloroplast stroma is transported to the central vacuole via RCBs, which are a type of autophagic compartment that specifically contains stromal proteins. The RCB pathway can facilitate the recycling of amino acids as an energy source. (**b**) When senescence is accelerated in individually darkened leaves, the active production of RCBs leads to chloroplast shrinkage, thereby allowing the transport of entire chloroplasts to the vacuole via chlorophagy. (**c**) Photodamage from exposure to ultraviolet-B (UV-B), strong visible light, or natural sunlight causes chloroplasts to collapse. The collapsed chloroplasts are then transported to the vacuole without prior activation of RCBs. This process is suggested to serve as a quality control mechanism that removes damaged chloroplasts.

During leaf senescence, the amount of chloroplast stromal proteins, including Rubisco, decreases prior to the reduction in the number of chloroplasts [33,34,35]. Therefore, stromal proteins appear to be degraded either inside or outside the chloroplast without the breakdown of the entire chloroplast. An immuno-electron microscopy (EM) analysis of Rubisco degradation in senescing wheat (*Triticum aestivum*) leaves revealed the presence of cytosol-localized small vesicles that contained Rubisco, but not thylakoid proteins such as light-harvesting chlorophyll a/b protein of Photosystem II (LHC II), α, β-subunits of coupling factor 1 in ATPase, or cytochrome *f* [36]. These vesicles, which are around 1 µm in diameter and are frequently surrounded by autophagosome-like double membranes, were originally referred to as Rubisco-containing bodies (RCBs). The development of live-cell imaging techniques using fluorescent protein markers allowed for the visualization of RCBs in vivo in Arabidopsis and rice (*Oryza sativa*) leaves expressing stroma-targeted green fluorescent protein (GFP) or GFP-labeled Rubisco [37,38]. This technique further demonstrated that RCBs are not produced in the mutant *atg5* or *atg7* lines, and that RCBs labeled with stroma-targeted red fluorescent proteins (RFPs) are co-localized with an autophagosomal marker, GFP-ATG8. These observations revealed that RCBs are a type of autophagic body that delivers a portion of the stromal proteins into the vacuole. Thus, the RCB pathway was established as an autophagic process that mobilizes stromal proteins to the vacuole (Figure 1a).

Endosomal sorting complex required for transport (ESCRT) proteins are part of an evolutionarily conserved system that is responsible for the remodeling of endosomal membranes in eukaryotes [39]. A recent study in Arabidopsis indicated that the ESCRT-III paralogs charged multivesicular body protein 1A (CHMP1A) and CHMP1B are required for the delivery of RCBs to the vacuole [40]. In *chmp1a chmp1b* double mutant plants, RCBs were produced but accumulated in the cytoplasm; therefore, CHMP1 proteins are required for the vacuolar sorting of chloroplast-derived RCBs or the fusion of autophagosomes enclosing RCBs. How a portion of stroma is separated as RCBs, and how RCBs are then recruited for autophagic transport remain unclear.

The RCB pathway is particularly active in sugar-starved, excised Arabidopsis leaves in darkness or the presence of photosynthesis inhibitors [41]. Starch is the major carbohydrate form for energy storage. The starchless mutants, *phosphoglucomutase* (*pgm*) and *ADP-glucose pyrophosphorylase1* (*adg1*), which lack starch, exhibited enhanced production of RCBs [41,42]. Moreover, starchless and *atg* double mutants exhibited reduced growth and enhanced cell death during developmental senescence compared to the respective single mutants [42]. These results indicate that the RCB pathway plays a role in the response to sugar starvation. Recent studies found that in the sugar-starved leaves of Arabidopsis plants maintained in complete darkness for several days, autophagy deficiency compromises the release of free amino acids, especially free branched chain amino acids (BCAAs) like isoleucine, leucine, and valine [43,44]. Arabidopsis mutants with defects in the enzymes involved in BCAA catabolism have reduced tolerance to sugar starvation due to prolonged complete darkness [9,45,46,47,48,49]; thus, BCAAs are a particularly important energy source for mitochondrial respiration as alternatives to sugars. The RCB pathway might supply free amino acids, especially BCAAs, derived from vacuolar degradation of stromal proteins as an alternative energy source during periods of impaired photosynthesis (Figure 1a).

Photosynthetic energy production can be perturbed by various types of suboptimal conditions, including shading, flooding, or drought. The importance of core autophagy machinery during submergence-induced hypoxia or draught stress was reported in Arabidopsis plants [50,51]. The RCB pathway might alleviate the energy limitation that is caused by some types of abiotic stresses.

RCB production is also activated during accelerated leaf senescence induced in leaves that were individually covered to impair photosynthesis [52]. This activation of senescence corresponds to chloroplast shrinkage. In addition to direct observations of RCBs labeled with stroma-localized fluorescent proteins, the activity of the RCB pathway can be monitored by biochemical detection of free GFP or RFP derived from vacuolar degradation of Rubisco-GFP or -RFP fusion proteins, which are mobilized to the vacuole via RCBs [53]. This technique indicated that autophagy contributes substantially to the degradation of Rubisco in individually darkened leaves and in those shaded by the leaves of neighboring Arabidopsis plants [53]. Such biochemical methods of monitoring the RCB pathway have also been established in rice plants, and have shown that Rubisco is degraded via RCBs in individually darkened rice leaves [38]. In autophagy-deficient *atg* mutant rice plants, *osatg7*, Rubisco degradation was attenuated in senescing leaves, which is consistent with the partly compromised nitrogen remobilization from lower leaves to newly developing upper leaves [54]. These findings further indicate that the RCB pathway mediates nitrogen remobilization from older leaves that cannot acquire sufficient light due to shading of developing leaves by upper tissues.

Analyses of Arabidopsis and maize (*Zea mays*) plants harboring the *atg* mutation indicated that autophagy contributes to nitrogen remobilization from vegetative tissues to reproductive tissues, including seeds [55,56,57]. However, such a role for autophagy in rice plants was not evaluated, because autophagy-deficient rice plants exhibit male sterility due to impaired pollen maturation [58].

## 3. Chlorophagy: Degradation of Entire Chloroplasts

Whereas the amount of stromal proteins decreases during the earlier stages of leaf senescence in wheat or barley (*Hordeum vulgare*) plants, the number of chloroplasts per cell decreases during the later stages [33,34,35]. In individually darkened leaves of wild-type Arabidopsis plants, RCB production and subsequent shrinkage of chloroplasts occur during the earlier stages of senescence, and the chloroplast population decreases during the later stages of senescence [52]. This decrease in chloroplast number is suppressed in *atg4* mutants. Some isolated vacuoles from the darkened leaves of wild-type plants contained chloroplasts that exhibited chlorophyll autofluorescence signals. These findings suggest that shrunken chloroplasts, which are produced through the active separation of their components in the RCB pathway, become the targets of autophagic transport as entire organelles, a process known as chlorophagy [59] (Figure 1b).

In yeast and mammals, autophagy is also recognized as a major quality control system for organelles through the selective removal of dysfunctional organelles [19]. In Arabidopsis *atg* plants, oxidized peroxisomes containing aggregated catalase accumulate in the cytoplasm of senescing leaves [60,61,62]. During germination, enzymes in peroxisomes catalyze β-oxidation and the glyoxylate cycle, thereby allowing lipids stored in seeds to be used as energy before photosynthetic machinery within chloroplasts are developed. As photosynthetic growth is established several days after germination, peroxisomes are remodeled to carry out the glycolate pathway, which is required for photorespiration. This functional conversion of peroxisomes was partly compromised in Arabidopsis *atg* plants in which peroxisome aggregates accumulate in mesophyll cells containing mature chloroplasts [63,64]. Thus, plant peroxisomes are likely targets of a process of selective autophagy known as pexophagy during senescence or seedling development. Autophagic degradation of the endoplasmic reticulum (ER) during ER stress due to tunicamycin treatment was also observed in Arabidopsis roots [65,66]. Selective degradation of ER by autophagy termed ER-phagy may function in plants.

A recent study investigated the involvement of autophagy in the turnover of chloroplasts under photooxidative stress conditions and demonstrated that chlorophagy is induced in Arabidopsis leaves damaged by UVB exposure [67]. A subset of the chloroplasts in the cytoplasm of UVB-damaged *atg5* and *atg7* plants exhibited irregular shapes and disorganized thylakoid structures. Chlorophagy was also induced by chloroplast damage caused by exposure to strong visible light or natural sunlight. Therefore, chlorophagy may remove entire photo-damaged chloroplasts by transporting them into the vacuole [67,68] (Figure 1c).

The chloroplast-targeted RCB pathway and chlorophagy differ in individually darkened leaves and in leaves subjected to UVB damage [67,69]. During sugar starvation in individually darkened leaves, RCBs were observed after 1 d of treatment, whereas chlorophagy was rarely observed during 3 days of dark treatment. By contrast, in leaves subjected to UVB-mediated oxidative stress, chlorophagy was actively induced 2 days after treatment without prior RCB production. These observations suggest that the induction of these two types of autophagy is individually controlled by distinct upstream mechanisms in response to environmental or developmental conditions (Figure 1).

## 4. ATI Body-Mediated Chloroplast Degradation

ATG8 is a core ATG protein that builds up the autophagosomal membrane by conjugating with phosphatidylethanolamine [70]. In yeast, several types of organelle-targeted autophagy are controlled by ATG proteins containing an ATG8-interacting motif (AIM) [71]. ATG32 triggers the removal of dysfunctional or excess mitochondria by interacting with autophagosomal membrane-anchored ATG8 on the mitochondrial outer envelope [72,73]. ATG39 and ATG40 were also identified as ATG8-interacting proteins that control nucleus- or ER-targeted selective autophagy, respectively [74].

**Figure 2:**
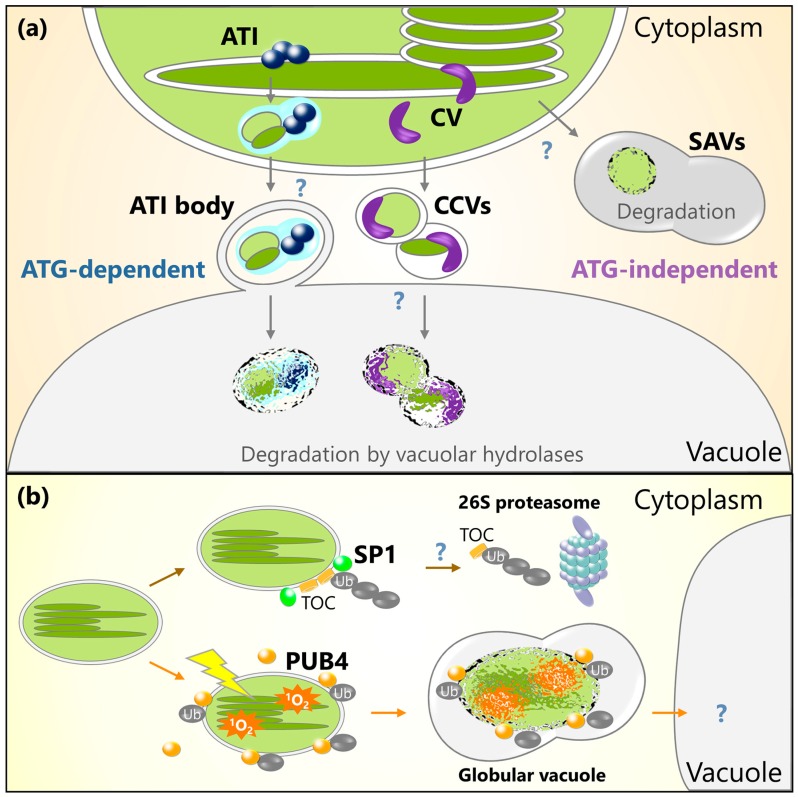
Schematic model for chloroplast protein turnover mediated by ATI bodies, CV-containing vesicles (CCVs), senescence-associated vacuoles (SAVs), or ubiquitination. (**a**) Plastid-associated ATI bodies are produced in chloroplasts and are then delivered into the central vacuole via an autophagy-dependent pathway. ATI bodies transport thylakoid, stroma, and envelope proteins. CV protein also interacts with thylakoid and stroma proteins, and then induces the production of CCVs that transport thylakoid, stroma, and envelope proteins into the central vacuole via an autophagy-independent pathway. SAVs are small lytic compartments that form in the cytoplasm. Stroma components are incorporated into the SAVs for digestion. (**b**) Chloroplast outer envelope-anchored E3 ligase, SP1, ubiquitinates TOC proteins and facilitates their degradation by 26S proteasome. Cytoplasmic E3 ligase PUB4 ubiquitinates oxidative chloroplasts accumulating ^1^O_2_ for the digestion of such chloroplasts in their entirety.

ATG8-interacting protein 1 (ATI1) and ATI2 were identified in a yeast two-hybrid screen for candidates that interact with the Arabidopsis ATG8 isoform, ATG8f [75]. These proteins were found to associate with plastids in addition to the ER as small vesicles of approximately 1 µm in diameter, which are referred to as ATI bodies [76]. A screen of potential ATI1-interacting proteins and microscopy observations of fluorescent marker proteins indicated that plastid-associated ATI bodies transport some thylakoid, stroma, and envelope proteins into the vacuole, especially under dark-induced energy limitation [76]. These delivery cargos differ from those of the RCBs that specifically contain a portion of stroma [36,37]; however, the vacuolar transport of plastid-associated ATI bodies is an autophagy-dependent process, as this body was not produced in the *atg5* mutants [76]. Therefore, ATI bodies represent a distinct form of autophagy vesicles that transport some stroma, thylakoid, and envelope components into the vacuole (Figure 2a). Plastid-associated ATI bodies are also observed inside the chloroplast, and ATI1 interacts with some thylakoid proteins in vivo [76]. It is thus proposed that plastid-associated ATI bodies form in chloroplasts and are then delivered into the vacuole via autophagosome-mediated transport (Figure 2a), although how such bodies are evacuated from chloroplasts remains unclear.

The appearance of plastid-associated ATI bodies in energy-starved seedlings or senescing leaves suggests that ATI bodies also contribute to amino acid recycling during starvation or senescence as part of the autophagy process, although the link between the induction level of the ATI bodies and changes in free amino acid content has not been evaluated. Additionally, plastid-associated ATI bodies are produced under salt stress, and ATI-knockdown plants have reduced salt tolerance [76]. The activation of autophagosome production and the reduced tolerance of *atg* mutants to salt stress were also observed in Arabidopsis plants [50,77]. These findings suggest that ATI bodies are involved in salt stress-induced chloroplast protein turnover.

## 5. Senescence-Associated Vacuoles

The formation of small, lytic senescence-associated vacuoles (SAVs) was reported when senescing leaves of Arabidopsis, soybean (*Glycine max*), and tobacco (*Nicotiana tabacum*) plants were stained with R-6502 dye, which emits strong fluorescence upon the hydrolytic activity of cysteine proteases [78,79]. Senescence-associated gene 12 (SAG12) is a senescence-induced cysteine protease localized within SAVs. SAVs are formed in the peripheral cytoplasmic region of mesophyll cells and are much smaller than the central vacuole, being approximately 0.7 µm in diameter. In addition, SAVs have greater lytic activity than the central vacuole and are strongly stained by lysotracker red or neutral red, fluorescent markers of acidic organelles.

SAV numbers increase as leaf senescence progresses [80]. Proteomic analysis of isolated SAVs in tobacco plants indicated that SAVs contain stromal proteins such as Rubisco and glutamine synthetase, but not thylakoid proteins such as LHCII and the reaction center D1 protein in photosystem II [79]. Treatment with a specific inhibitor of cysteine proteases, E-64, partially suppressed Rubisco degradation in the tobacco leaf discs [80]. These observations suggest that SAVs contribute to senescence-induced Rubisco degradation, similar to RCBs; however, *atg7* mutants produced SAVs [79]. Therefore, SAVs may be an autophagy-independent, extra-chloroplastic route for the degradation of stromal proteins in senescent leaves (Figure 2a). How stromal proteins are transported into the SAVs remains uncertain.

## 6. Autophagy-Independent Vesicles Derived from Chloroplasts

The *chloroplast vesiculation* (*CV*) gene encodes a plastid-targeted protein in rice plants that is strongly upregulated under abiotic stress and downregulated by cytokinin [81]. In Arabidopsis, expression of the *CV-GFP* construct under the control of the dexamethasone-inducible promoter caused the formation of a type of chloroplast-derived vesicle exhibiting strong CV-GFP signal referred to as CV-containing vesicles (CCVs) [81]. CCVs are around 1 µm in diameter and contain stroma, envelope, and thylakoid proteins, as demonstrated by immunoblot analysis of some chloroplast proteins, co-immunoprecipitation assays of potential CV-interacting protein, and confocal microscopy of fluorescent marker proteins of chloroplast stroma [81]. CCVs do not associate with the autophagosome marker GFP-ATG8a, and the *atg5* mutation does not affect the production of CCVs. Additionally, CCVs do not associate with SAVs stained with lysotracker red. Thus, CCVs are part of a vacuolar degradation process for chloroplasts that is independent of autophagy and SAVs (Figure 2a).

Immuno-EM analysis showed that CV-GFP was associated with the thylakoid or envelope membranes before CCV production [81]. The interaction of CV with PsbO protein, a subunit in the thylakoid-bound photosystem II complex, was confirmed by co-immunoprecipitation detection and a bimolecular fluorescence complementation (BiFC) assay. These results indicate that CV interacts with some proteins inside the chloroplast before CCVs form. The C-terminal domain of CV, which is largely conserved among CV orthologs of various plant species, is required for CCV production [81]; however, how chloroplast-targeted CV induces the formation of CCVs and chloroplast destabilization has not been evaluated.

In Arabidopsis plants, endogenous *CV* was upregulated in senescing leaves and leaves subjected to oxidative stress or salt stress [81]. Consistent with this, transient expression of *CV* caused accelerated leaf senescence, and the suppression of *CV* transcript by miRNA led to increased leaf longevity under salt stress. Similarly, in rice plants, the RNAi silencing of *CV* expression led to delayed leaf senescence under water deficit stress, and the transient overexpression of *CV-GFP* under the control of the β-estradiol-inducible promoter accelerated leaf senescence symptoms [82]. Elevated *CV* transcript levels were observed in UVB-damaged Arabidopsis leaves [67]. *CV* may activate the destabilization and degradation of chloroplasts through the formation of CCVs during senescence, especially under stress conditions in Arabidopsis and rice plants.

## 7. Ubiquitin E3 Ligase-Associated Chloroplast Degradation

The ubiquitin proteasome system (UPS) is an evolutionarily conserved major protein degradation system in eukaryotic cells [83,84,85]. During UPS-mediated proteolysis, the polypeptide ubiquitin acts as a sorting signal for the degradation of specific proteins by the 26S proteasome in the ubiquitination cascade. Ubiquitin is activated by E1 proteins and then transferred to E2 ubiquitin conjugating enzymes. The transfer of ubiquitin from E2s to target proteins requires E3 ubiquitin ligases. The resulting ubiquitinated proteins are selectively incorporated into the 26S proteasome complex for breakdown. Eukaryotic genomes generally encode a large family of E3s and Arabidopsis plants can theoretically express more than 1500 of these proteins [86,87,88]. The ubiquitination of specific proteins by individual E3s allows for highly controlled, selective protein degradation by the UPS.

The UPS was shown to contribute to the degradation of chloroplast proteins in an experiment using suppressor of ppi1 locus 1 (SP1) isolated from Arabidopsis plants [89]. SP1 is a chloroplast outer envelope-anchored E3 ligase that ubiquitinates some proteins of the translocon on the outer chloroplast membrane (TOC) complex (Figure 2b). Most nucleus-encoded chloroplast proteins are imported into chloroplasts through the TOC and translocon on the inner chloroplast membrane (TIC) complexes [90]. During the greening of etiolated seedlings, etioplasts, which are a type of plastid present in non-green tissues, are converted to mature chloroplasts; therefore, large amount of photosynthetic proteins encoded in the nuclear genome are expressed and imported into the plastid via TIC-TOC complexes. *sp1* mutant plants exhibit delayed maturation of chloroplasts during the greening of etiolated seedlings [89]. Thus, SP1 likely serves as a control for protein import into chloroplasts via the turnover of the TOC complex when etioplasts develop into functional chloroplasts. *sp1* mutants also showed delayed leaf yellowing during dark-induced accelerated senescence; conversely, SP1-overexpressing plants showed an enhanced decline of photosynthetic efficiency [89]. SP1-mediated TOC turnover may further regulate protein import into chloroplasts when functional chloroplasts are actively degraded during senescence.

SP1 induces the degradation of TOC during oxidative stress caused by salt or osmotic stress, thereby attenuating protein import into the chloroplasts [91]. Under these stress conditions, accumulation of hydrogen peroxide (H_2_O_2_), a type of ROS, was enhanced in *sp1* mutants and was alleviated in SP1-overexpressing plants. SP1-mediated degradation of the TOC complex by UPS suppresses photosynthetic activity and thereby limits ROS production [91], since ROS are produced during photosynthesis. SP1-mediated TOC turnover may therefore control the chloroplast proteome and photosynthetic capacity in response to stress. It is still unclear how ubiquitinated proteins on outer-envelope proteins are solubilized to allow degradation by UPS localized in the cytoplasm.

A recent study reported that a cytosol-localized E3 ligase functions in the degradation of entire chloroplasts [92]. When dark-germinated, etiolated seedlings of the Arabidopsis mutant of plastid *ferrochelatase 2* (*fc2*) are transferred from darkness to light, their chloroplasts over-accumulate singlet oxygen (^1^O_2_), thereby leading to the death of photosynthetic cells and compromised greening of plants. A suppressor mutant (referred to as *pub4–6* [92]) of this inhibited greening phenomenon had an amino acid substitution in *plant u-box 4* (*PUB4*), which encodes a cytosolic ubiquitin E3 ligase. Although EM analysis indicated that entire chloroplasts were digested in the cytoplasm during compromised greening in *fc2* plants, this degradation of chloroplasts was lower in *fc2 pub4–6* plants, even though ^1^O_2_ accumulation was not suppressed. Therefore, PUB4-related ubiquitination triggers the digestion of entire chloroplasts that are accumulating ^1^O_2_ (Figure 2b). However, unlike the *pub4–6* mutation, the T-DNA insertional knockout mutations of *PUB4* (referred to as *pub4–1* and *pub4–2*) did not suppress the phenotype of the *fc2* mutant during greening [92]. Therefore, it is unclear how PUB4 is involved in the ubiquitination of ^1^O_2_ accumulating chloroplasts and their subsequent degradation.

In mammalian cells, ubiquitination largely acts as a trigger of autophagic removal of dysfunctional organelles [93]. During mitophagy, depolarized mitochondria are ubiquitinated by the E3 ligase Parkin, allowing for the autophagic removal of targeted mitochondria into the lysosome [94,95,96,97]. During the greening of *fc2* mutants, some chloroplasts appeared to be degraded in the cytoplasm, and the interaction between degrading chloroplasts and the vacuole via a globule-like structure was observed [92]. Such observations were distinct from the vacuolar chloroplasts that result from chlorophagy in leaves exposed to strong visible light (1200–2000 µmol·m^−2^·s^−1^), where entire chloroplasts exhibiting thylakoid membranes are localized in the central vacuole in EM imaging [67]. Furthermore, the *pub4–6* and *atg10* mutants are phenotypically distinct, as *atg10* plants showed accelerated senescence during dark treatment compared to wild-type plants, but *pub4–6* plants did not [92]. Therefore, PUB4-related ubiquitination is unlikely a simple trigger of autophagy. 

## 8. Future Perspectives

Our understanding of the diverse extraplastidic pathways mediating chloroplast protein degradation has progressed in the past decades. Table 1 compares their relationships to core autophagy machinery, plant species, induction stimuli, and degradation targets. It is clear that multiple pathways are induced during diverse stress conditions, such as sugar starvation, senescence, and oxidative stress (Table 1). Thus, new questions about chloroplast turnover arise, including why plants have multiple processes for chloroplast protein turnover, and how these processes are differentially utilized. Future research should examine how extraplastidic systems are coordinated with intraplastidic proteolysis. The RCB pathway is activated during the earlier stages of dark treatment and chlorophagy is induced during the later stages [52,67], suggesting that several pathways are induced at distinct time points during leaf senescence and stress responses. An important role of intrachloroplastic proteases in chloroplast protein turnover during photodamage was largely demonstrated [15,16]. Therefore, extraplastidic pathways that are induced during photooxidative stress might be triggered when intraplastidic proteolysis is insufficient for maintaining chloroplast functions.

In Arabidopsis *atg* plants, both *CV* expression and proteasome activity are increased [43,98], suggesting a complementary relationship among some of the chloroplast-associated degradation systems. However, since senescence symptoms are largely accelerated in *atg* mutants due to the over-accumulation of salicylic acid [99], the increase in *CV* expression or proteasome activity in *atg* plants can also be interpreted as a result of accelerated senescence and cell death. To better understand the process of chloroplast protein turnover and to decipher the relationships among the diverse pathways mediating this process, the mechanisms regulating these pathways will need to be elucidated. It would be fascinating to determine whether the distinct pathways that mediate chloroplast degradation share a common upstream regulatory mechanism, or whether they are regulated independently. In addition, how small vesicles delivering portions of chloroplasts, including RCBs, ATI bodies, and CCVs, are derived from entire chloroplasts largely remains to be explained.

The extraplastidic routes for chloroplast protein turnover were largely identified using Arabidopsis plants (Table 1). This advance greatly expanded our understanding of chloroplast protein turnover in important cereals, such as rice and maize [38,54,56,82]. Chloroplast degradation is strongly linked to nitrogen remobilization and the changes of photosynthetic capacity that are important determinants of productivity in crop plants. Therefore, manipulating chloroplast protein turnover might be an effective strategy to improve the productivity of crops. In rice plants, RNAi-mediated silencing of *CV* led to an increase in grain yield under water deficit stress [74]. Studies showed that Arabidopsis plants overexpressing one of the core *ATGs* had an enhanced stress tolerance [100,101]. In addition, SP1-overexpressing Arabidopsis plants had improved tolerance to oxidative stress [91]. Therefore, elucidating the molecular basis of multiple processes for chloroplast protein turnover in Arabidopsis plants may suggest strategies to improve the productivity and stress tolerance of crop plants.

## Figures and Tables

**Table 1:** List of extraplastidic degradation pathways described.

Pathway	Relationship to Core Autophagy Machinery	Analyzed Species	Degradation Targets	Stimuli ^b^	References
RCBs (Rubisco-containing bodies)	dependent	Arabidopsis, rice, wheat	stroma, envelope	sugar starvation, senescence	[36,37,38,41,42,44,52,53,54]
Chlorophagy	dependent	Arabidopsis	entire chloroplasts	photodamage, senescence	[52,67]
ATI bodies	dependent	Arabidopsis	stroma, thylakoid, envelope	sugar starvation, salt stress, senescence	[76]
SAVs (Senescence-associated vacuoles)	independent	Arabidopsis, soybean, tobacco	stroma	senescence	[78,79,80]
CCVs (Chloroplast vesiculation-containing vesicles)	independent	Arabidopsis, rice	stroma, thylakoid, envelope	senescence, salt stress, oxidative stress	[81,82]
E3 ligase SP1	- ^a^	Arabidopsis	TOC proteins on outer envelope	senescence, greening, oxidative stress	[89,91]
E3 ligase PUB4	- ^a^	Arabidopsis	entire chloroplasts	Oxidative stress (^1^O_2_)	[92]

^a^ The link of the E3 ubiquitin ligases to autophagy has not been directly examined. ^b^ Stimuli inducing the respective pathways.
